# Strategies for drug repurposing against coronavirus targets

**DOI:** 10.1016/j.crphar.2021.100072

**Published:** 2021-12-04

**Authors:** Poppy O. Smith, Peiqin Jin, Khondaker Miraz Rahman

**Affiliations:** School of Cancer and Pharmaceutical Sciences, King's College London, Franklin-Wilkins Building, 150 Stamford Street, London, SE1 9NH, UK

**Keywords:** Coronavirus, SARS-CoV-2, Drug repurposing, *In silico* screening

## Abstract

Repurposing regulatory agency approved drugs and investigational compounds with known safety profiles can significantly fast track the drug development timeline over *de novo* drug discovery, with lower investment requirements and improved attrition rate. These advantages are vital in any epidemic or pandemic situation, where hospital beds are occupied by patients for whom there is no known treatment. Here we examine drug repurposing in the context of human coronaviruses, SARS-CoV, MERS-CoV, and, in particular, SARS-CoV-2, the virus currently causing a continued widespread pandemic with substantial impacts on public health and economy. The key druggable targets explored were those involved in viral entry, viral replication, and viral-induced ARDS, as well as viral proteases, with a focus on the strategy by which the drugs were repurposed.

## Abbreviations

3CL^pro^: 3-chymotrypsin-like protease
ACE2: angiotensin-converting enzyme 2
AI: artificial intelligence
ARDS: acute respiratory distress syndrome
ASM: acid sphingomyelinase
BALB: Bagg albino (mouse stain)
CC_50_: half maximal cytotoxic concentration
CoV: coronavirus
COVID-19: Coronavirus disease 2019, formerly ‘2019 novel coronavirus’ (2019-nCoV)
CQ: chloroquine
CT: computerised tomography
EC_50_: half maximal effective concentration
EUA: emergency use authorisation
FDA: Food and Drug Administration
FIASMA: functional inhibition of acid sphingomyelinase
H1N1: hemagglutinin type 1 and neuraminidase type 1 (influenza strain)
HCoV: human coronavirus
HCQ: hydroxychloroquine
HCV: hepatitis C virus
HIV: human immunodeficiency virus
IC_50_: half maximal inhibitory concentration
IFN: interferon
IL: interleukin
MERS-CoV: Middle East Respiratory Syndrome coronavirus
N protein: nucleocapsid protein
NIH: National Institutes of Health
NSP: non-structural protein
PL^pro^: papain-like protease
RdRp: RNA-dependent RNA polymerase
ReFRAME: Repurposing, Focused Rescue, and Accelerated Medchem
RSV: respiratory syncytial virus
S protein: spike protein
SARS-CoV: Severe Acute Respiratory Syndrome coronavirus
SARS-CoV-2: Severe Acute Respiratory Syndrome coronavirus 2
TMPRSS2: transmembrane protease/serine subfamily member 2
WHO: World Health Organisation

## 1 Introduction

**Fig. 1:**
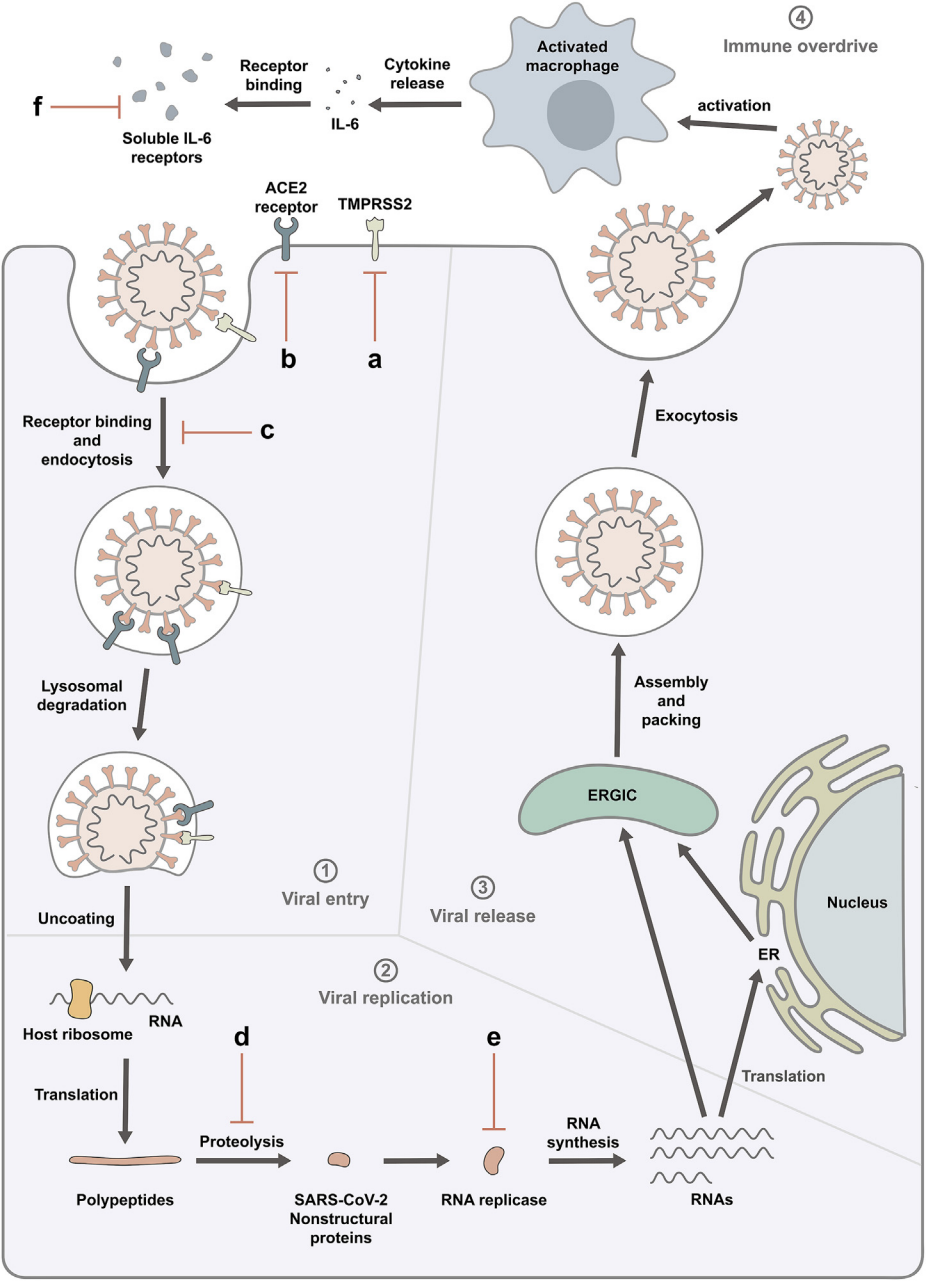
**SARS-CoV-2 Viral Lifecycle and Drug Targets**. (**1**) HCoV virus enters host cells by recognition of viral spike protein by angiotensin-converting enzyme 2 (ACE2) receptor - inhibited by (**b**) chloroquine, and hydroxychloroquine and (**c**) umifenovir - causing conformational change in S protein, exposing protease cleavage site acted upon by transmembrane protease/serine subfamily member 2 (TMPRSS2) - inhibited by (**a**) camostat, and nafamostat - resulting in membrane fusion of viral particle and host cell. Alternatively, the viral particle is endocytosed proceeding to a lysosome in which the S protein is cleaved by cathepsin L, triggering fusion of the lysosome membrane with the viral particle. (**2**) Once the viral RNA genome is released into the host cell, it is translated by the host ribosome to give polypeptides. These polypeptides undergo proteolysis by viral proteases papain-like protease (PL^pro^) and 3-chymotrypsin-likease protease (3CL^pro^) – inhibited by (**d**) lopinavir in combination with ritonavir – to give viral proteins, such as non-structural proteins (NSPs), including the RNA-dependent RNA polymerase (RdRp), also known as RNA replicase – inhibited by (**e**) remdesivir, ritonavir, and favipiravir. The RNA replicase copies the viral genome. (**3**) The copies of the viral genome are processed for viral release by host machinery translation, the endoplasmic reticulum (ER) and the endoplasmic-reticulum-Golgi intermediate compartment (ERGIC). New viral particles are assembled and released from the host cell by exocytosis. (**4**) Presence of the viral particles in the host cause immune overdrive, activating immune responses, including the production of cytokines, such as interleukin-6 (IL-6) – effects of which are inhibited by (**f**) tocilizumab, and sirolimus. IL-6 triggers the cytokine storm which induces acute respiratory distress syndrome (ARDS) – modulated by corticosteroids, such as dexamethasone and prednisolone, interferons (IFNs), and interferon-inducers, such as nitazoxanide. Adapted from (Sanders et al., 2020).

Coronaviruses (CoVs) are positive-sense, single-stranded RNA, enveloped viruses belonging to the *Coronaviridae* family, of the order *Nidovirales*. Based on their genome sequence, four genera of CoVs exist; α, β, γ, and δ, of which α- and β-CoVs can infect humans. There are 7 human coronaviruses (HCoVs), four (HCoV-OC43, HCoV-HKU1, HCoV-229E, and HCoV-NL63) cause 15% of common colds and self-limiting upper respiratory tract infections in non-immunocompromised patients. The remaining 3 HCoVs (Severe Acute Respiratory Syndrome Coronavirus (SARS-CoV), Middle East Respiratory Syndrome Coronavirus (MERS-CoV), and Severe Acute Respiratory Syndrome Coronavirus 2 (SARS-CoV-2)) cause severe, possibly fatal, respiratory diseases with viral pneumonia as widespread epidemics or, in the case of SARS-CoV-2, a pandemic. The outbreak of SARS-CoV in 2003 affected 5 continents with a fatality rate of 10%. Later, in 2012, MERS-CoV spread across the Arabian Peninsula with a 35% fatality rate. SARS-CoV-2, originating in 2019 Wuhan, spread across all 7 continents with over 181 million cases and over 3.9 million deaths as of June 28, 2021 by the John Hopkins University Center for Systems Science and Engineering, giving the virus a 2.2% fatality rate but far higher transmission than previous CoV epidemics (Cascella et al., 2021; Elshabrawy, 2020; Y. Huang, Yang et al., 2020; Wu et al., 2020a, Wu et al., 2020b). Due to the heightened pathogenicity, high fatality rates, and pandemic potential of SARS-CoV, MERS-CoV and SARS-CoV-2, repurposed drugs against these 3 HCoVs was the focus here. Druggable targets within the SARS-CoV-2 lifecycle explored here are summarised in Fig. 1, with a focus on drugs with SARS-CoV-2 *in vitro* efficacy ≤100 ​μM EC_50_, with active or completed clinical trials by ClinicalTrials.gov as of March 2021.) (see

Drug repurposing, also known as drug repositioning, is the branch of drug discovery which identifies alternative uses for regulatory approved drugs or clinical-stage compounds with known pharmacological and safety profiles, drastically accelerating their drug development timeline over *de novo* drug design. Drug repurposing is especially valuable under time pressured scenarios, such as pandemics, where hospital beds are occupied by patients for whom there is no known treatment. The ∼10-year time frame of *de novo* drug design is incompatible with pandemic timescales. Here the fast-track nature of drug repurposing becomes invaluable (Yadi Zhou, Hou, et al., 2020). Most pandemics are viral (Morse et al., 2012), with the most recent being the ongoing coronavirus disease 19 (COVID-19) pandemic caused by SARS-CoV-2. Three broad strategies have been identified for drug repurposing under pandemic situations: 1) exploring existing broad-spectrum antiviral drugs; 2) screening molecular databases of approved or clinical-stage compounds for molecules with potential antiviral therapeutic effects; and 3) utilising an artificial intelligence (AI), network-based technology to examine the virus-host interactome, identifying potential repurposing candidates that target virus-host interactions (Wu et al., 2020a, Wu et al., 2020b; Yadi Zhou, Hou, et al., 2020). Strategies 1) and 2) are the most frequently employed, with the merits of strategy 3) becoming ever more apparent with its increasing use (D. E. Gordon et al., 2020; Riva et al., 2020; Yadi Zhou, Hou, et al., 2020; Yadi Zhou, Wang, Tang, Nussinov, & Cheng, 2020).

## 2 Repurposing strategies targeting viral entry

Targeting viral entry into host cells can be achieved by inhibiting various points in the process, including membrane fusion through S protein-host receptor interaction, host proteases, and endolyososmal pathway. As viral entry is the first step in the virus life cycle, its inhibition is considered favourable because viral damage to the host cell is minimised, as is the opportunity for acquisition of viral resistance (Laws et al., 2020; Yamamoto et al., 2016).

### 2.1 Membrane fusion inhibitors

**Fig. 2:**
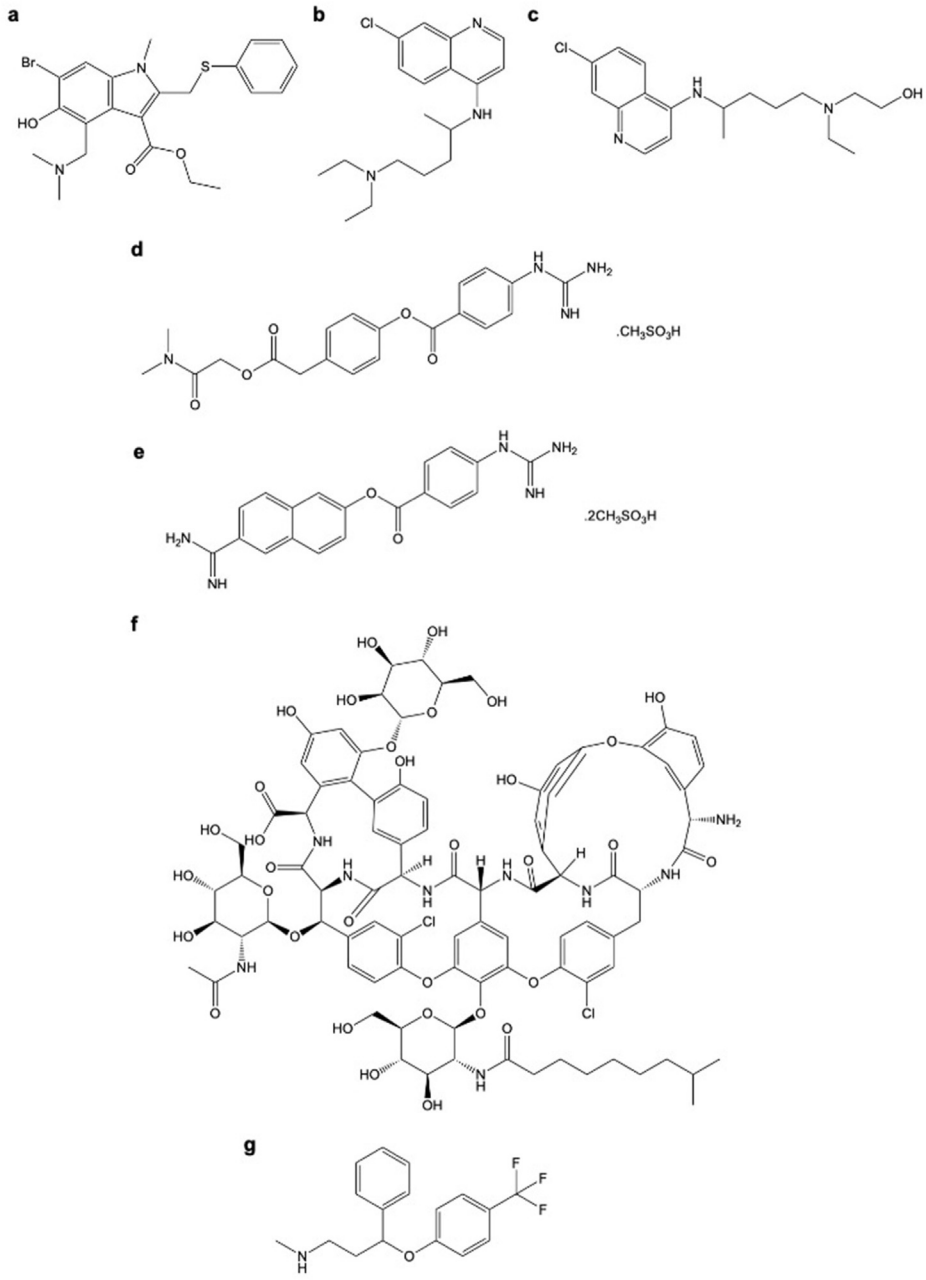
**Chemical Structure of Viral Entry Inhibitors** (**a**) umifenovir, (**b**) chloroquine, (**c**) hydroxychloroquine, (**d**) camostat, (**e**) nafamostat, (**f**) teicoplanin, and (**g**) fluoxetine.

Disrupting membrane fusion between the host cell and viral particle can be achieved through inhibitor binding to the S protein, preventing recognition by host receptors. Of the most promising inhibitors, umifenovir (Arbidol®), chloroquine (Aralen®) (CQ) and hydroxychloroquine (Plaquenil®) (HCQ) (Fig. 2) have been clinically evaluated, however, systemic reviews and meta-analyses showed no clinical benefit was observed (Huang et al., 2021). Umifenovir was evaluated against HCoV by homology modelling and target-based screening against the ZINC Drug Database, identifying umifenovir to most favourably interact with the S protein with a −145.125 mfScore (Chaomin Wu et al., 2020). The potential activity of umifenovir against HCoVs was also explored by Touret et al. (2020), using umifenovir as a control against the screening of the Prestwick Chemical Library of approved drugs in SARS-CoV-2 infected VeroE6 and Caco-2 ​cells, reporting an EC_50_ of 10.7 ​μM with a low cytotoxicity (CC_50_ ​> ​40 ​μM). Umifenovir is approved for use in Russia and China against influenza-induced respiratory tract infections with no serious adverse effects and has reported *in vitro* activity against a range of viruses, including influenza A H1N1, respiratory syncytial virus (RSV), adenovirus, and rhinovirus, hence the reasoning for its screening in HCoVs (Chaomin Wu et al., 2020). In addition to inhibiting viral entry, umifenovir can also induce interferon (IFN) production (Lian et al., 2020). In 2004, Masterlek™ patented the use of umifenovir against SARS-CoV-induced pneumonia (Blaising et al., 2014). Later, in 2016, umifenovir was patented as an anti-MERS-CoV medicine (CN106074506A). However, as mentioned above, umifenovir has shown no clinical benefit against SARS-CoV-2 (Huang et al., 2021).

Modification of the host receptor complementary to the S protein provides an alternative approach to membrane fusion inhibition. CQ and HCQ, originally approved for, but not limited to, treatment of malaria, alter the glycosylation of angiotensin-converting enzyme 2 (ACE2), the S protein-binding receptor in SARS, thereby indirectly interfering with the S protein-host receptor interaction in SARS-CoV and SARS-CoV-2. CQ and HCQ were investigated for their use against coronaviruses due to their broad-spectrum antiviral activity and use in human immunodeficiency virus (HIV) (Devaux et al., 2020; Fantini et al., 2020; Laws et al., 2020). Further to interfering with S protein-ACE2 interaction, the multifaceted antiviral mechanism of CQ/HCQ also includes endosome alkalisation preventing the pH-dependent activation of cathepsin L, viral protein post-translational modification disruption, and anti-inflammatory response mediation through cytokine reduction; therefore the drugs also inhibit MERS-CoV replication (Devaux et al., 2020). However, as with umifenovir, no clinical benefit of CQ/HCQ against SARS-CoV-2 was found, with HCQ associated with an increased mortality rate (Axfors et al., 2021).

Greater success has been seen with repurposed function inhibition of acid sphingomyelinase (FIASMA) medications to reduce SARS-CoV-2 infection of host cells. Acid sphingomyelinase (ASM) catalyses the formation of ceramide, resulting in ceramide-rich membrane domains which gather multiple ACE2 receptors, facilitating viral entry (Carpinteiro et al., 2020; Hoertel et al., 2021a, Hoertel et al., 2021b; Le Corre and Loas, 2021). Amitriptyline, amlodipine, and emetine, along with other FIASMAs, were evaluated against HCoVs through various *in silico*, *in vivo* and *ex vivo* models summarised by Le Corre & Loas (Le Corre and Loas, 2021). The use of FIASMA medications against COVID-19 has been associated with reduced incidence of intubation and death in clinical studies (Darquennes et al., 2021; Hoertel et al., 2021a, Hoertel et al., 2021b).

### 2.2 Host protease inhibitors

Transmembrane protease/serine subfamily member 2 (TMPRSS2), cathepsin L and furin are key host proteases implicated in the proteolytic processing of CoV which is essential for viral entry, hence their inhibition should attenuate viral infection (Artika et al., 2020; Elshabrawy, 2020; Laws et al., 2020; J. Zhang et al., 2020). The S protein priming by TMPRSS2 was found to be crucial for SARS-CoV viral spread and the preferred entry route, over endocytosis, for SARS-CoV and MERS-CoV (Hoffmann et al., 2020a, Hoffmann et al., 2020b; Iwata-Yoshikawa et al., 2019). Camostat (Foipan® or camostat mesylate), a serine protease inhibitor approved in Japan for chronic pancreatitis and postoperative reflux esophagitis (Yanchen Zhou et al., 2015), was found to be active against TMPRSS2 in MERS-CoV through a high throughput, cell-based, dual split reporter protein assay giving good *in vitro* activity (1 ​μM IC_50_) (Yamamoto et al., 2016). *In vitro* activity of camostat against SARS-CoV, MERS-CoV, and SARS-CoV-2 infected Calu3 cells has been subsequently reported (Hoffmann et al., 2021; Hoffmann et al., 2020a, Hoffmann et al., 2020b). With regards to *in vivo* activity, camostat gave a 60% survival rate in BALB/c mice lethally infected with SARS-CoV (Yanchen Zhou et al., 2015). The preliminary clinical effectiveness of camostat was observed in a small retrospective cohort study (Choi et al., 2020).

The Yamamoto et al. screen also identified regulatory-approved pancreatitis drug and anticoagulant, nafamostat (Futhan® or nafamostat mesylate), as a potent TMPRSS2 inhibitor, achieving MERS-CoV fusion inhibition at one-tenth of the concentration of camostat (0.1 ​μM IC_50_) (Yamamoto et al., 2016). Moreover, nafamostat was predicted to have a low binding energy with SARS-CoV-2 TMPRSS2 in a computational docking study of 36 molecules (Rensi et al., 2020). *In vitro* activity of nafamostat against SARS-CoV (1.4 ​nM EC_50_), MERS-CoV (5.9 ​nM EC_50_), and SARS-CoV-2 (5.0 ​nM EC_50_) in Caclu3 cells were reported by Hoffmann et al. (Hoffmann et al., 2020). Clinical trials of nafamostat are ongoing (as of March 2021 by ClinicalTrials.gov).

Whilst TMPRSS2 may be the primary host protease involved in viral entry, simultaneous inhibition of TMPRSS2 and cathepsin L is more efficacious, with the potential to completely block viral entry (Hoffmann et al., 2020a, Hoffmann et al., 2020b; Kawase, Shirato, van der Hoek, Taguchi and Matsuyama, 2012; Shirato et al., 2013), hence inhibitors of cathepsin L are also required. Teicoplanin, a glycopeptide antibiotic used to treat Gram-positive bacterial infections with low toxicity (Zhang et al., 2020), was tested against MERS-CoV and SARS-CoV in a cell-based, high throughput screening assay of Topscience's Food and Drug Administration (FDA)-approved drug library of 1600 compounds with *in vitro* activity in HEK293T cells (IC_50_: 0.63 ​μM MERS-CoV, 3.76 ​μM SARS-CoV) (Zhou et al., 2016). In 2020, it was found that teicoplanin inhibited *in vitro* SARS-CoV-2 entry in A549 ​cells (1.66 ​μM IC_50_) with concentration far below the routine clinical human blood concentration (8.78 ​μM for 400 ​mg daily dose). Teicoplanin was also effective at inhibiting SARS-CoV-2 entrance into HEK293T and Huh7 cells (Zhang et al., 2020). Teicoplanin was patented for the treatment of MERS-CoV in 2016 (WO2016201692A1) but results of COVID-19 clinical trials are pending.

The MERS-CoV and SARS-CoV-2 S proteins contain a furin cleavage site. Furin cleavage facilitates binding of a higher proportion of S proteins to the host receptor by pre-activating the S protein for TMPRSS2 cleavage (Hoffmann et al., 2020a, Hoffmann et al., 2020b; Johnson et al., 2021). Loss of the cleavage site reduced virus replication in VeroE6 cells and attenuated pathogenesis in hamster and mouse models (Johnson et al., 2021), illustrating the importance of furin activity despite no repurposed drugs against furin in active or completed clinical trials.

### 2.3 Endolysosomal pathway inhibitors

The endosome into which the viral particle is endocytosed presents a host-pathogen interface for drug targeting (Schloer et al., 2020; Yang and Shen, 2020). Fluoxetine, a FIASMA medication as well as a selective serotonin reuptake inhibitor (SSRI) antidepressant, has been proposed to impair endolysosomal acidification and cholesterol accumulation of in late endosomes/lysosomes, disrupting the endolysosomal host-pathogen interface (Schloer et al., 2020). Fluoxetine was repurposed against SARS-CoV-2 by screening against infected Vero cells and observation of its effectiveness against incoming influenza A virus cells, giving EC_50_ ∼1 ​μM with CC_50_ ∼40 ​μM (Schloer et al., 2020; Zimniak et al., 2021). Fluoxetine has been shown to significantly reduce COVID-19 deterioration (Hoertel et al., 2021a, Hoertel et al., 2021b). However, the general success of repurposing antidepressants against COVID-19 may be limited due to antidepressants typically displaying low therapeutic indices, resulting in their micromolar range *in vitro* efficacies translating to significantly higher doses than therapeutically accepted (Ferguson, 2001; Khushboo and Sharma, 2017).

## 3 Repurposing strategies targeting viral replication

The non-structural proteins (NSPs) are the most conserved proteins across all CoVs and are involved in essential viral lifecycle steps. Arguably, RNA-dependent RNA polymerase (RdRp) (also known as NSP12) and helicase NSP13 are two of the four most vital NSPs due to their roles in replication, hence their inhibition is an attractive antiviral target (Totura and Bavari, 2019). Other approaches to interfering with viral replication exist, such as inhibition of nuclear import (Caly et al., 2020; Wagstaff et al., 2012).

### 3.1 RNA-dependent RNA polymerase (RdRp) inhibitors

**Fig. 3:**
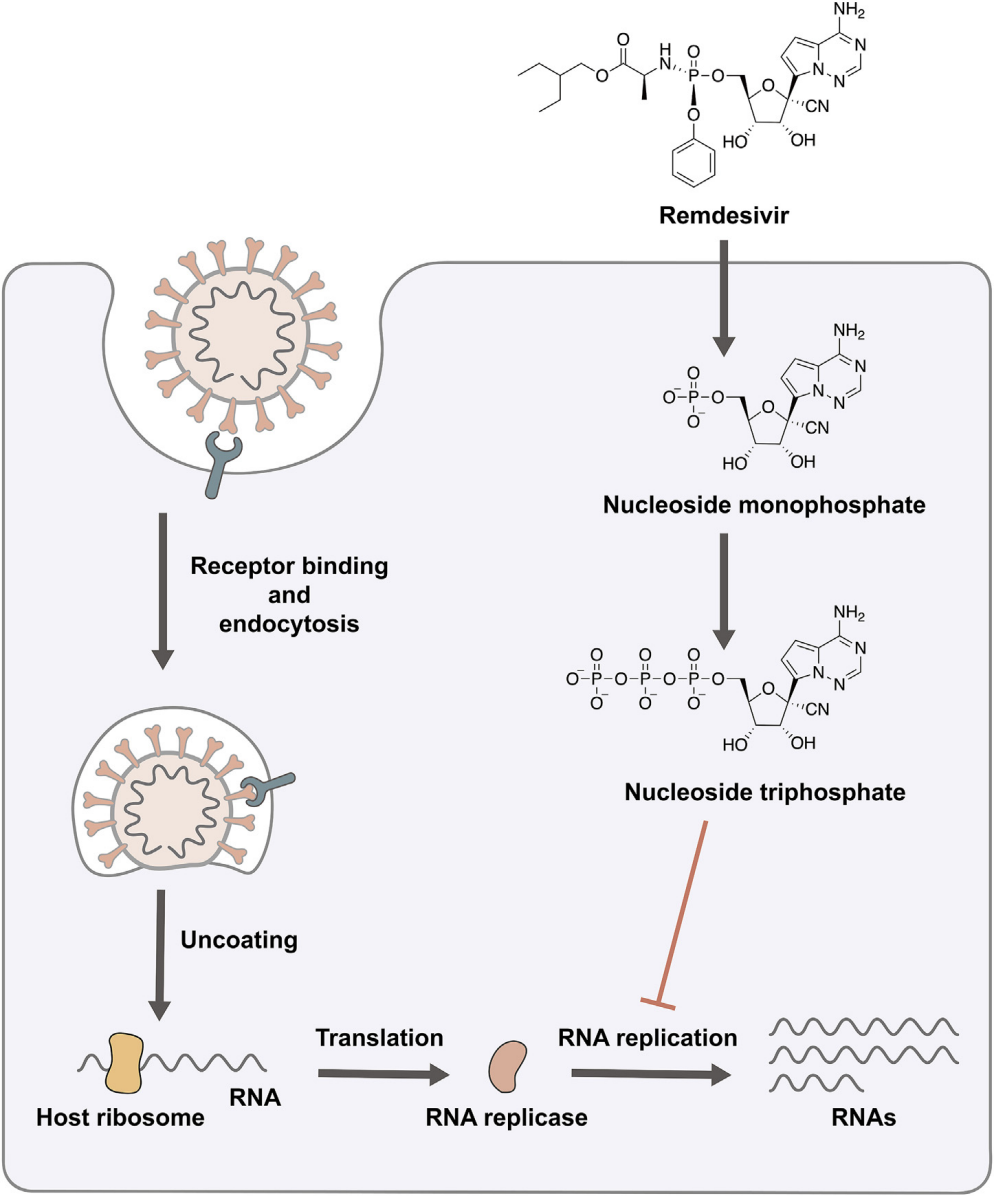
**Remdesivir Mechanism of Action**. Upon entering the host cell, remdesivir is converted to its nucleoside triphosphate via its nucleoside monophosphate. The nucleoside triphosphate inhibits RNA replication by acting as a nucleotide analogue, stalling RNA synthesis after the addition of three more nucleotides.

Remdesivir (Veklury®), an investigational new drug originally developed against HIV and RSV (Lo et al., 2017; Malin et al., 2020) identified as the most promising anti-CoV therapeutic, targets RdRp (Kupferschmidt, 2020; Sanders et al., 2020; Sheahan et al., 2017) (Fig. 3). Part of the attraction to targeting RdRp is its absence in humans, therefore inhibition should not cause significant host toxicity (Chaomin Wu et al., 2020; Canrong Wu et al., 2020). Remdesivir has the added advantage of resisting exoribonuclease removal (Agostini et al., 2018). The first clinical use of remdesivir was against Ebola rendering the virus undetectable after the treatment course, with no adverse clinical or biochemical effects recorded (Jacobs et al., 2016). Whilst remdesivir was not previous regulatory-approved, due to its clinical trial success (NCT04280705) and established safety profile (Jacobs et al., 2016), FDA granted remdesivir emergency use authorisation (EUA) against SARS-CoV-2 on May 1, 2020. Soon after, on May 7, 2020, remdesivir was approved in Japan as the first SARS-CoV-2 treatment and then on October 22, 2020 by the FDA for use in against COVID-19 in hospitalised patients over 12 years, weighing ≥40 ​kg. Amongst others, remdesivir was evaluated against HCoV by Elfiky (2020) through molecular docking studies against SARS-CoV-2 RdRp, yielding a binding energy of −7.6 ​kcal/mol for remdesivir. Wang et al. (2020) identified remdesivir through standard cell assays as a potent anti-SARS-CoV-2 in infected Vero E6 (0.77 ​μM EC_50_, >100 ​μM CC_50_) and Huh7 cells. Touret et al. (2020) used remdesivir as a validation control in their screen of the Prestwick Chemical Library against SARS-CoV-2 infected VeroE6 and Caco-2 ​cells, reporting remdesivir *in vitro* activity (1.6 ​μM EC_50_) and illustrating 100% replication inhibition at 5 ​μM. Clinical studies on remdesivir have shown treatment significantly reduces COVID-19 recovery time with remdesivir treatment a frontline HCoV therapeutic (Beigel, Tomashek, & Dodd; Grein et al., 2020; Maffei and Sonia, 2020; Wang et al., 2020).

The promising anti-CoV activity of remdesivir led to the investigation for additional HCoV therapies with enhanced efficacy by Riva et al. (2020), including synergistic relationships with the Repurposing, Focused Rescue, and Accelerated Medchem (ReFRAME) library, of ∼12,000 regulatory-approved, clinical trialled or significantly characterised compounds, with remdesivir. The high throughput cell-based screen identified 4 compounds with dose-dependent activities which gave synergy at notable levels with remdesivir: hanfangchin A, SB-616234-A, MLN-3897, and VBY-825 (Riva et al., 2020).

The importance of remdesivir's resistance to exoribonuclease removal is seen with another RdRp inhibitor, ribavirin (Rebetol®, Virazole® and Copegus®) (Fig. 5). Ribavirin, an FDA-approved hepatitis C virus (HCV) treatment, was tested against CoV by Elfiky (2020) through molecular docking studies against SARS-CoV-2 RdRp of direct-antiviral drugs, yielding a binding energy of −7.8 ​kcal/mol, the tightest binding of the 5 approved drugs explored. However, the *in vitro* doses required to inhibit SARS-CoV, MERS-CoV and SARS-CoV-2 replication in Vero cells (∼100 ​μM EC_50_), exceed attainable concentrations in humans due to excision of ribavirin from RdRp nucleotide active site by CoV proofreading mechanisms (Barnard et al., 2006; Totura and Bavari, 2019; Wang et al., 2020). However, *in vitro* potency of ribavirin appears to be cell line dependent as Morgenstern et al. (2005) reported different ribavirin *in vitro* activities for MA104 (9.4 ​μM EC_50_), Caco2 (7.3 ​μM EC_50_), HPEK (5.2 ​μM EC_50_), and PK-15 (2.2 ​μM EC_50_) SARS-CoV infected cells, despite ribavirin giving no *in vitro* inhibition in Vero cells. This may explain the comparatively low *in vitro* efficacy observed in SARS-CoV-2 infected Vero E6 cells (109.5 ​μM EC_50_) (Wang et al., 2020). Nevertheless, ribavirin showed no activity against SARS-CoV mouse models, instead exacerbating disease (Barnard et al., 2006; Day et al., 2009). However, in combination with IFNs, as is done in HCV treatment (Barnard et al., 2006), ribavirin showed *in vitro* activity at concentrations applicable to the clinic (12 ​μM IC_50_ for ribavirin with 125 U/mL IFN-α2b against MERS-CoV infected Vero E6 cells; 0.3 ​μM EC_50_ for ribavirin with 28 U/mL IFN-β against SARS-CoV infected Caco2 cells) (Falzarano et al., 2013a, Falzarano et al., 2013b), reduced viral replication and moderated host response such that breathing abnormalities and pneumonia did not develop in MERS-CoV primate models (Falzarano et al., 2013a, Falzarano et al., 2013b; Morgenstern et al., 2005). All ClinicalTrials.gov clinical investigations into ribavirin for SARS-CoV-2 involve a combinatorial approach, as of March 2021, with completed trials only reporting significant effectiveness of ribavirin against COVID-19 in combination with IFN (Hung et al., 2020; Tong et al., 2020).

Favipiravir (Avigan®), first approved for the treatment of neuraminidase inhibitor resistant influenza in Japan, potently and selectively targets RdRp enzymes of RNA viruses through inhibiting its RNA polymerase activity and thus is effective against a wide range of RNA viruses and is often used to treat resistant strains (Furuta et al., 2017). Favipiravir was repurposed against SARS-CoV-2 by Wang et al. (2020) due to its well-known broad spectrum antiviral activity, and by Chaomin Wu et al. (2020) in their homology modelling and target-based screening against the ZINC Drug Database, despite the docking scores of favipiravir being relatively low. The *in vitro* activity of favipiravir against SARS-CoV-2 infected cells is disputed: Wang et al. (2020) reported a modest *in vitro* activity (61.88 ​μM EC_50_, >400 ​μM CC_50_) in Vero E6 cells, Shannon et al. (2020) reported weak activity (207.1 ​μM EC_50_), whilst Choy et al. (2020) reported no evident antiviral effect at concentrations <100 ​μM, and Jeon et al. (2020) observed no antiviral activity at all. Driouich et al. (2021), using a SARS-CoV-2 Syrian hamster model, demonstrated pre-emptive or preventive administration of favipiravir at moderate and high doses significantly reduced infection titres (moderate: *P* ​≤ ​0.038; high: *P* ​< ​0.0001), and clinically alleviated disease compared with no treatment. The efficacy of favipiravir was improved using a preventive approach, suggesting favipiravir may be more suited to prophylactic use. However, signs of toxicity were observed at the high doses, therefore using favipiravir in combination with other antivirals should be investigated to lower dosing. A similar study by Kaptein et al. (2020) supported the *in vivo* antiviral potency of high favipiravir doses. The FDA cleared favipiravir as an investigational new drug allowing it to proceed to US clinical trials (Gallagher, 2020), and the Drug Controller General of India granted favipiravir emergency approval for mild to moderate COVID-19 infections, with approval also granted in Saudi Arabia and the UAE (Agrawal et al., 2020). The clinical benefit of favipiravir remains to be validated, as of March 2021, by comparison to placebo (Cai et al., 2020; C. Chen et al., 2020; Dabbous et al., 2021; Lou et al., 2021).

### 3.2 Helicase NSP13 inhibitors

During replication, NSP13 is responsible for the unwinding of double-stranded viral RNA, a role previously identified as indispensable, making NSP13 a necessary component of CoV replication (Chaomin Wu et al., 2020). Nevertheless, repurposing of NSP13 inhibitors is limited (Chaomin Wu et al., 2020). The bananin derivatives of adamantane, used as antivirals and muscle relaxants, were identified through a chemical genetic approach against SARS-CoV by Tanner et al. (2005) with good *in vitro* activity (0.5–3 ​μM IC_50_) and low toxicity (>300 ​μM CC_50_). The SARS-CoV-2 homology modelling and virtual ligand screening of Chaomin Wu et al. (2020) against the ZINC Drug Database identified promising mfScores for regulatory-approved lymecycline, cefsulodine, rolitetracycline, itraconazole, saquinavir, dabigatran, and canrenoic acid binding to NSP13. However, few NSP13 inhibitors progress past the preclinical stage due to the inhibition of host ATPases and kinases by helicase inhibitors which target the NSP13 ATPase activity (Elshabrawy, 2020). Additionally, promising mfScores do not guarantee efficacious preclinical activity, as seen with itraconazole, a drug with one of the best mfScores, failing to reduce viral load and prevent transmission in a SARS-CoV-2 hamster model (Liesenborghs et al., 2021).

### 3.3 Alternative viral Replication Inhibitors

**Fig. 4:**
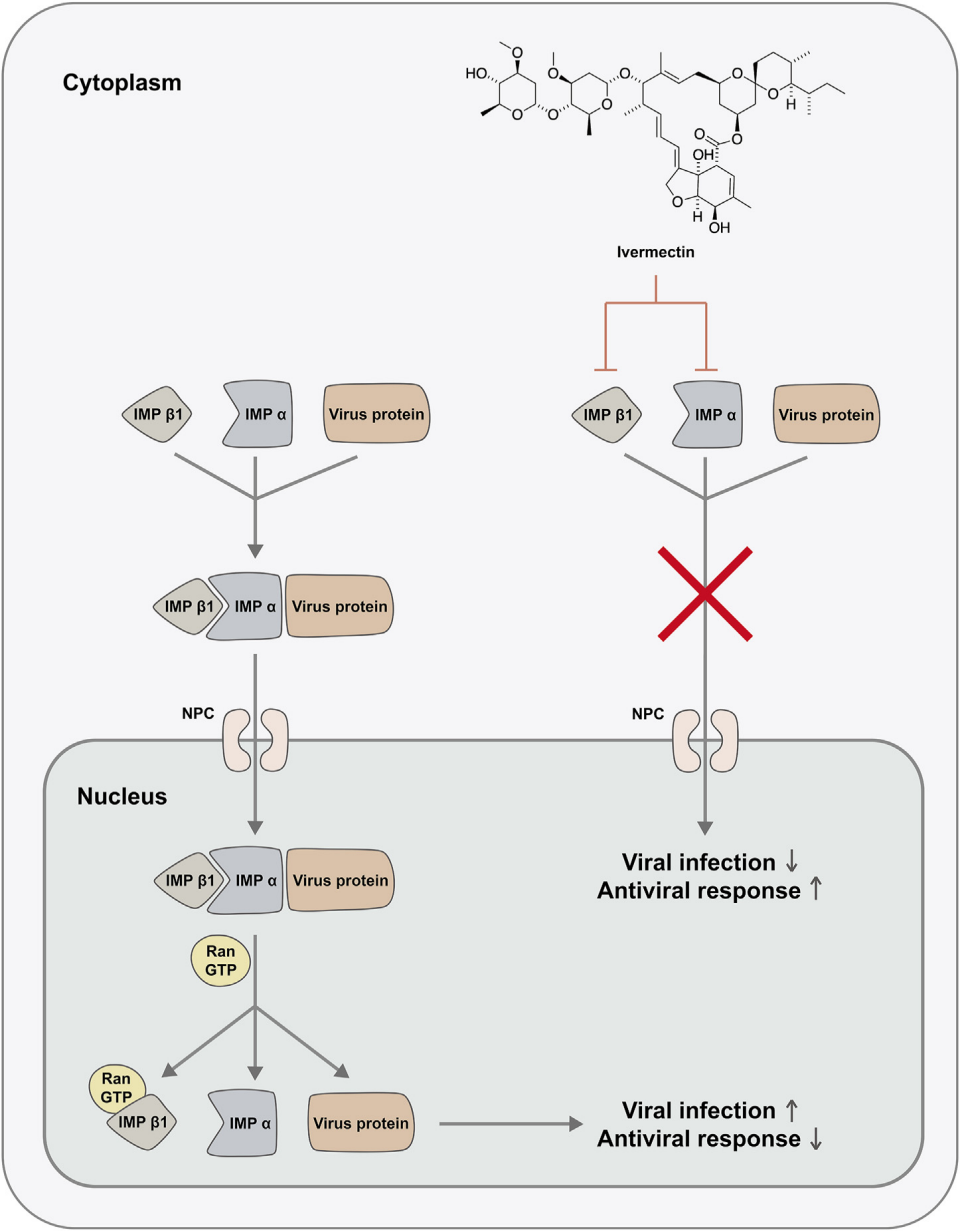
**Proposed Mechanism of Action for Replication Inhibitors targeting Nuclear Import**, exemplified with the suggested mechanism of action for ivermectin, inhibiting the formation of the importin (IMP) α/β1 heterodimer with the viral protein, preventing nuclear import which would increase viral infection and decrease host antiviral response.

Despite RNA virus replication occurring in the cytoplasm of the host cell, a variety of RNA viruses rely on nuclear import mediated by the host importin (IMP) α/β1 heterodimer during infection for viral replication and survival (Yang et al., 2020). Inhibition of IMPα/β1-mediated nuclear import, through IMPα/β1 dissociation or inhibiting the complex formation, has been shown to reduce viral replication and viral load in HIV-1, dengue virus and influenza (Wagstaff et al., 2012). As CoVs are hypothesised to also utilise this IMPα/β1-mediated nuclear import, regulatory-approved drugs inhibiting this mechanism have been repurposed against SARS-CoV-2 (Caly et al., 2020; Maurya, 2020). One such drug is ivermectin, approved as an anti-parasitic against nematode infections, such as onchocerciasis and scabies (Wagstaff et al., 2012), evaluated against HIV-1 using an AlphaScreen®-based high throughput screening assay of 480 compounds selected from the Library of Pharmacologically Active Compounds (LOPAC) (Wagstaff et al., 2011). Ivermectin has been one of the most controversial repurposed drugs against COVID-19, widely used throughout the US and Latin America despite its lack of clinical benefit and toxicity (Davey, 2021; Peña-Silva et al., 2020). Caly et al. (2020) proposed ivermectin would prevent SARS-CoV-2 protein nuclear import by binding to host IMPα/β1 (Fig. 4). They also reported efficacious *in* *vitro* activity against SARS-CoV-2 infected Vero-hSLAM cells (2.5 ​μM IC_50_). This activity was supported by molecular docking and dynamic simulations of ivermectin with a variety of SARS-CoV-2 and host proteins, with significant binding affinity which would negatively impact viral entry and replication. This study additionally proposed doxycycline, an FDA-approved synthetic antibiotic, to be used in combination with ivermectin (Maurya, 2020). However, Momekov and Momekova (2020) showed, from analysing ivermectin dosing regimens and maximal plasma concentrations in parasitic diseases, the concentrations required to inhibit SARS-CoV-2 are 50- to 100-fold higher than those attainable in humans without significant toxicity. de Melo et al. (2021) demonstrated, using golden Syrian hamster as a model for COVID-19, whilst ivermectin did not reduce viral load, clinical deterioration was prevented and they suggested ivermectin was acting as an immunomodulator, significantly reducing cytokine IL-6:IL-10 ratio in the lungs. Such immunomodulatory effects are believed to be due to ivermectin acting as a positive allosteric modulator of the α-7 receptor involved in cytokine repression (de Melo et al., 2021; Zdanowski et al., 2015). Therefore, ivermectin may be more useful targeting the immune system effects of CoVs opposed to viral replication specifically (DiNicolantonio et al., 2020). However, as eluded to earlier, no clinical benefits of ivermectin against SARS-CoV-2 have been identified (Chaccour et al., 2021) and its use for COVID-19 has been recommended against by the FDA and NIH (FDA, 2021; NIH, 2021a).

**Fig. 5:**
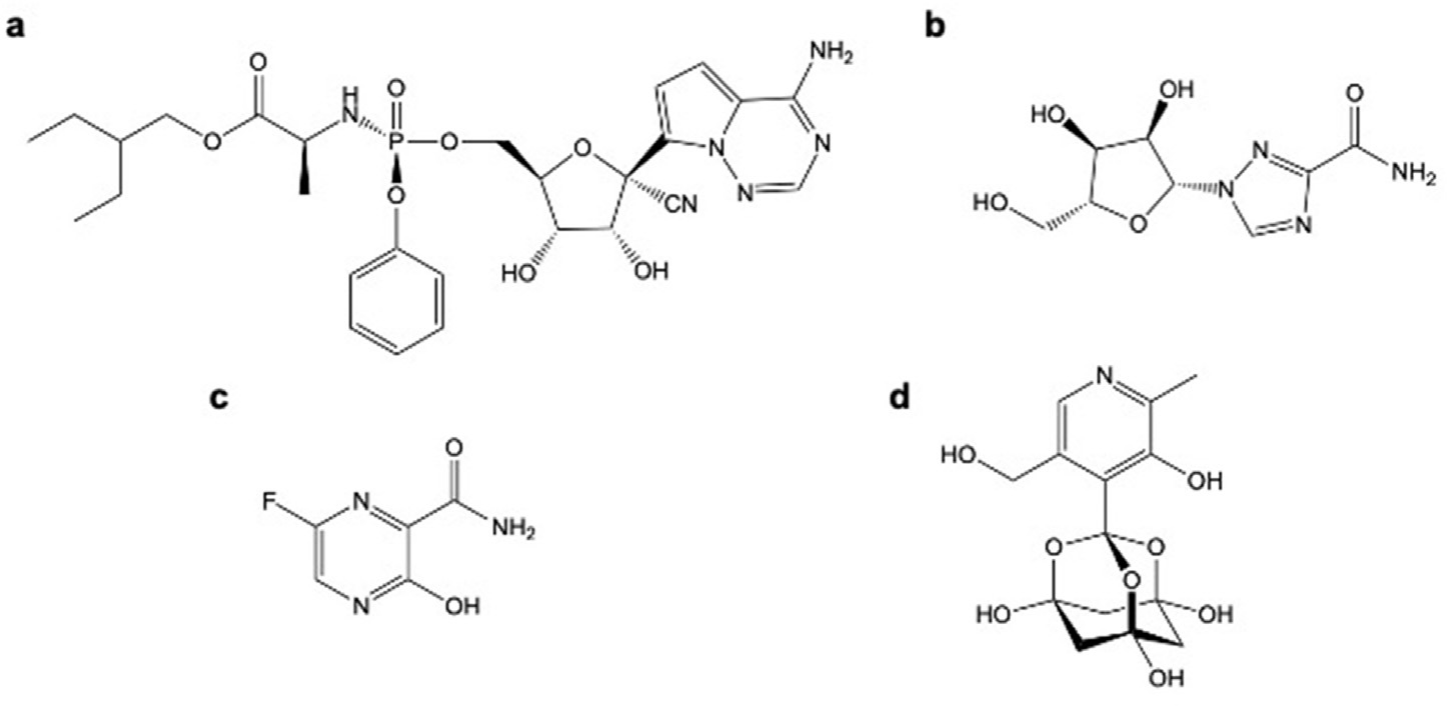
**Chemical Structure of Viral Replication Inhibitors** (**a**) remdesivir, (**b**) ribavirin, (**c**) favipiravir, and derivatives of (**d**) bananin.

## 4 Repurposing strategies targeting viral proteases

Other than RdRp and helicase NSP13, the other two most vital NSPs are 3-chymotrypsin-like protease (3CL^pro^) and papain-like protease (PL^pro^) for the essential proteolytic processing they perform in the CoV lifecycle (Totura and Bavari, 2019). Therefore, it is unsurprising both have been used to screen for CoV inhibitors (Chaomin Wu et al., 2020).

### 4.1 3-Chymotrypsin-like protease (3CL^pro^) inhibitors

**Fig. 6:**
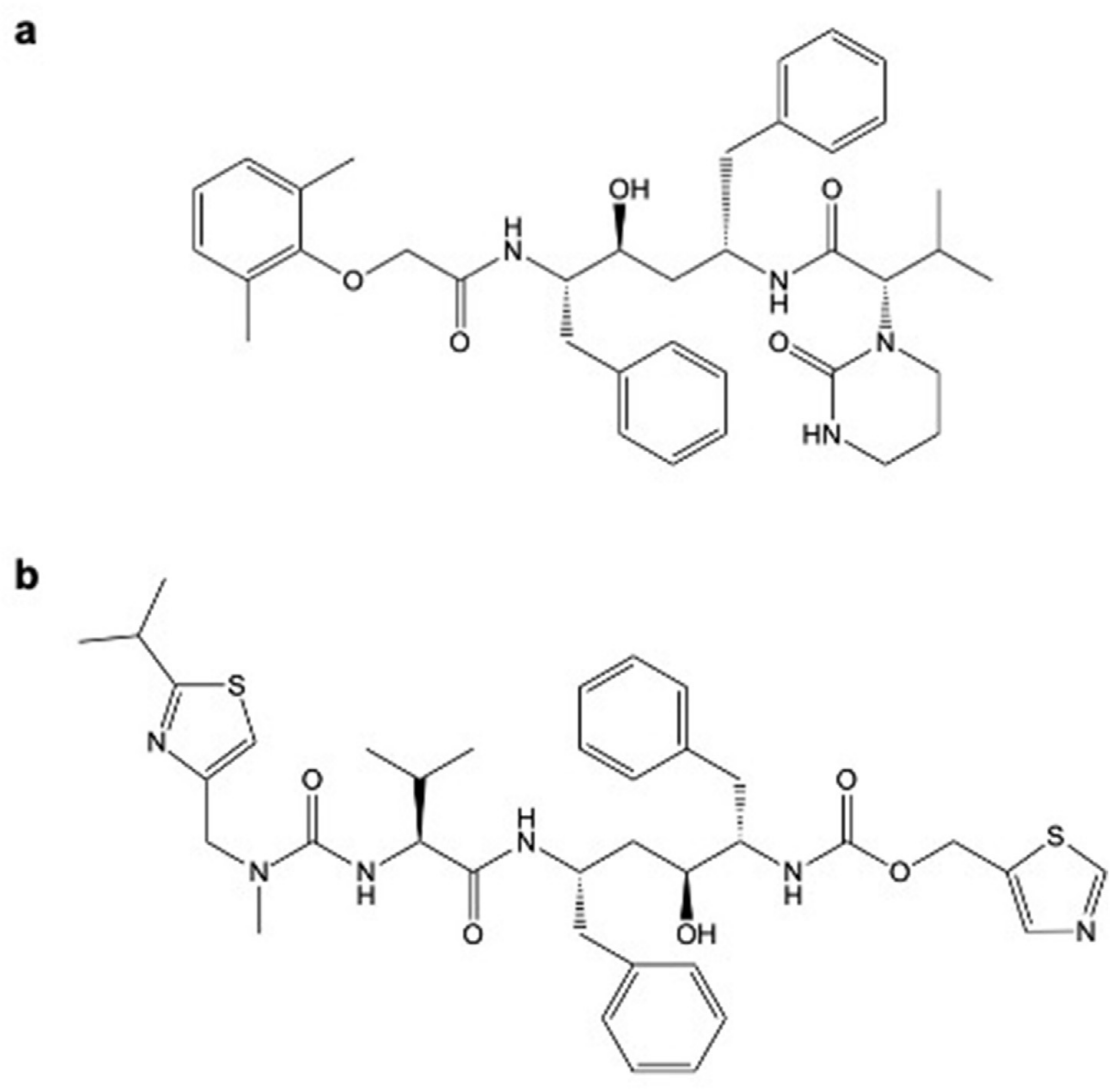
**Chemical Structure of Viral Protease Inhibitor** (**a**) lopinavir administered with (**b**) ritonavir.

The essentiality of 3CL^pro^ is exemplified by its direct mediation of NSPs maturation, including facilitating the formation of major viral proteins RdRp and NSP13, thus the attraction to its inhibition in the treatment of HCoVs (Cherian et al., 2020; Chaomin Wu et al., 2020). Lopinavir, was first evaluated through a Vero cell culture screening assay of 348 FDA-approved compounds against SARS-CoV and MERS-CoV replication inhibition (De Wilde et al., 2014), giving activity at reasonable concentrations (EC_50_: 17.1 ​μM SARS-CoV, 8.0 ​μM MERS-CoV; CC_50_: >32 ​μM SARS-CoV, 24.4 ​μM MERS-CoV) despite showing inactivity in other studies (Sheahan et al., 2020). The virtual screen of 7173 purchasable drugs against a 3D molecular model of SARS-CoV-2 3CL^pro^ conducted by Chen, Yiu and Wong (2020) also identified lopinavir, with a binding affinity higher than the mean score. Lopinavir was shown to have a SARS-CoV-2 *in vitro* activity of 26.6 ​μM EC_50_ in Vero E6 cells (Choy et al., 2020). The *in vivo* study examining the therapeutic effect of lopinavir/ritonavir (Fig. 6) in combination with IFN-β1b against MERS-CoV in common marmosets, reported the combinatorial treatment gave improved clinical outcomes and significantly lowered viral load in the lungs (*P* ​= ​0.036) compared to the untreated group; concluding the combination should be evaluated in clinical trials (Chan et al., 2015). Despite there being only 3 animals per group in this study, it is the only *in vivo* study examining lopinavir/ritonavir against MERS-CoV. No *in vivo* studies of lopinavir/ritonavir have been conducted against SARS-CoV (Yao et al., 2020). The use of lopinavir/ritonavir for treatment of hospitalised COVID-19 patients was discontinued in February 2021 after trial data showed no clinical benefit (Consortium, 2021; Horby et al., 2020; NIH, 2021b).

### 4.2 Papain-like protease (PL^pro^) inhibitors

NSP1-3 are 3 essential CoV proteins required for correcting viral replication. PL^pro^ is vital for the generation of these NSPs, its protease activity releasing each of the 3 products from the replicase polyprotein which forms all CoV NSPs (Harcourt et al., 2004). PL^pro^ also has confirmed significance in the antagonisation of the host immune response. Therefore, PL^pro^ has been ruled indispensable for successful CoV infection (Chaomin Wu et al., 2020). Thus, unsurprisingly, PL^pro^ has been used as a target in screening studies of regulatory-approved drugs against SARS-CoV-2; including Kouznetsova et al. (2020) who performed data mining of a ∼2500 compound FDA-approved drug database, followed by docking against a protein-based pharmacophore model of SARS-CoV-2 PL^pro^ binding pocket. This resulted in a featured list of 59 docked drugs, 2 of which, valganciclovir and pemextred, were also identified by Chaomin Wu et al. (2020) in their homology modelling and target-based screening against the ZINC Drug Database. Chaomin Wu et al. (2020) also identified ribavirin, thymine, chloramphenicol, cefamandole, tigecycline, chlorphenesin carbamate, and levodropropizine to have high binding affinity with PL^pro^. The homology model and *in silico* docking study of Arya et al. (2020), which screened 2525 FDA-approved drugs from the DrugBank database and ZINC15 library, identified one of the same drugs as Kouznetsova et al., chloroquine, alongside 15 others of which biltricide, cinacalcet, procainamide and terbinafine had the highest binding affinities. Other than those already covered in this review, all of the featured drugs from these computational studies remain in preclinical development, with no active or completed clinical trials.

## 5 Repurposing strategies targeting acute respiratory distress syndrome (ARDS)

**Fig. 7:**
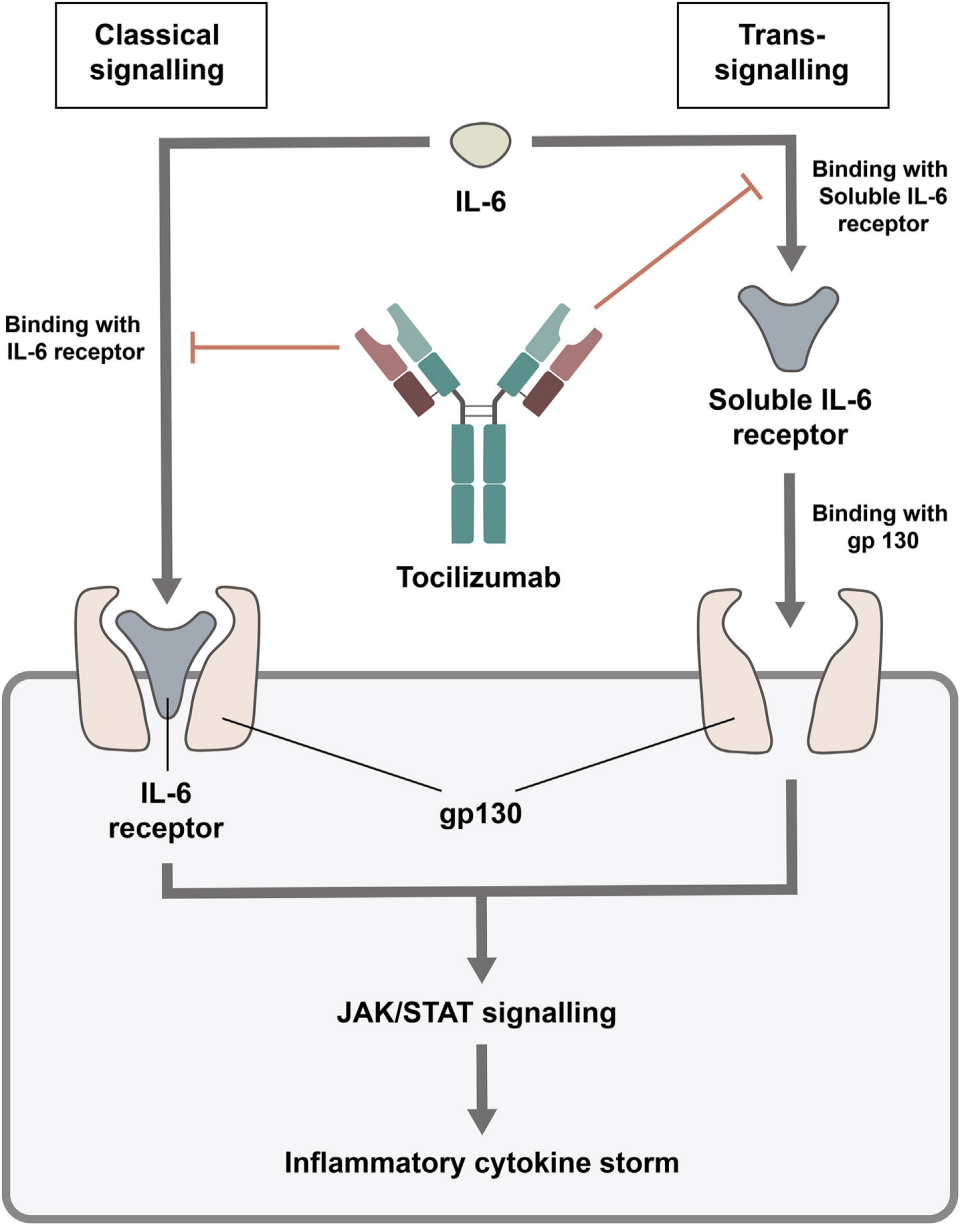
**Tocilizumab Mechanism of Action**. Tocilizumab prevents the induction of cytokine storm by inhibiting the binding of interleukin-6 (IL-6) by competitive binding to membrane-bound and soluble IL-6 receptors.

**Fig. 8:**
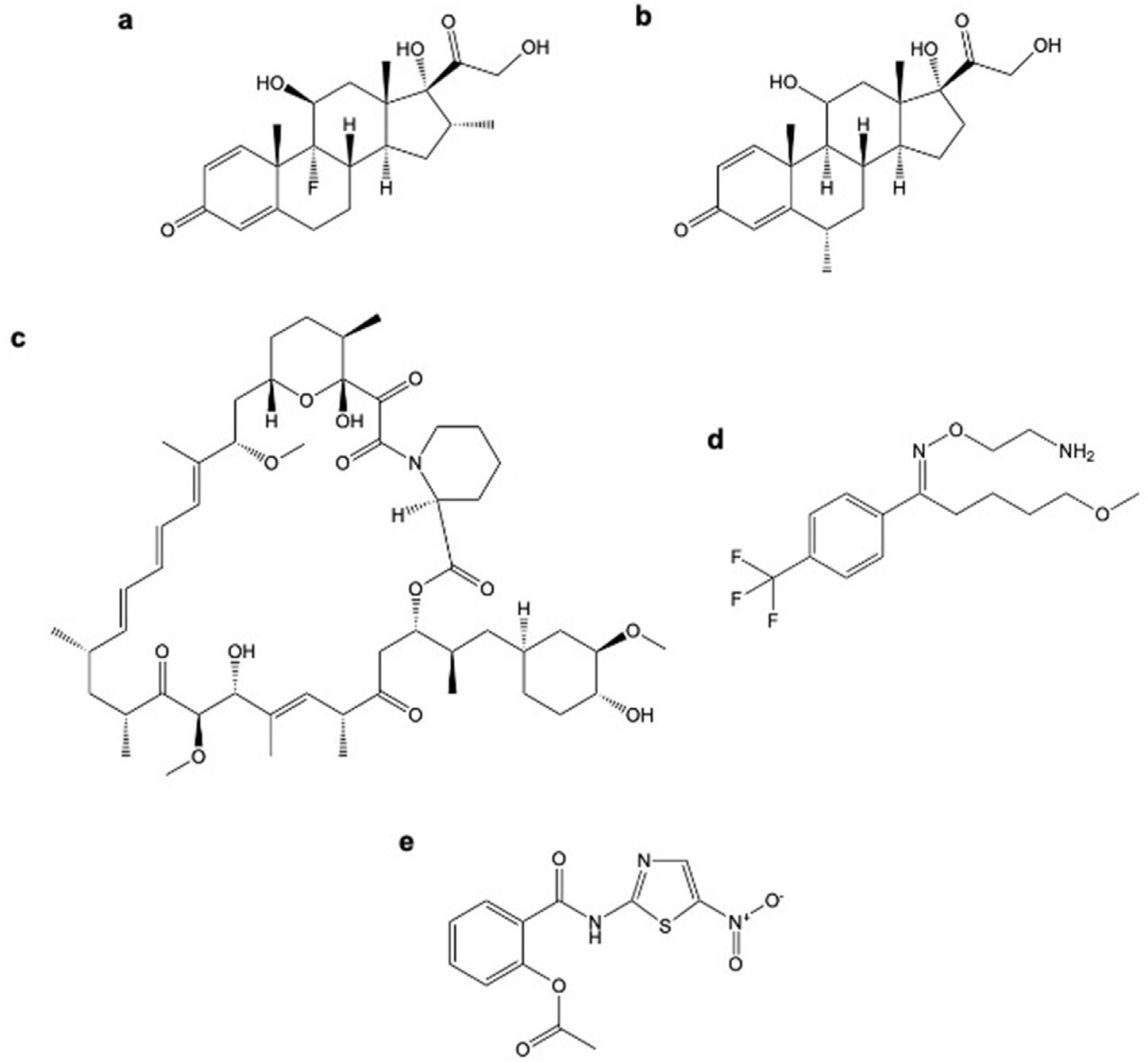
**Chemical Structure of Acute Respiratory Distress Syndrome (ARDS) Inhibitors**, (**a**) dexamethasone, (**b**) methylprednisolone, (**c**) sirolimus, (**d**) fluvoxamine, and (**e**) nitazoxanide.

The main cause of death in SARS-CoV-2 cases is acute respiratory distress syndrome (ARDS), induced by dysregulation of the host immune response, leading to cytokine storm and abnormal inflammatory responses (Chen et al., 2020; Shi et al., 2020; Wang et al., 2020). This dysregulation is involved in HCoV replication and infection control (Omrani et al., 2014). Therefore, modulating this response by treating the immunopathology of infection could decrease HCoV ARDS-associated mortality (Cao, 2020). Approaches to inhibit ARDS includes the use of anti-inflammatory drugs and immunomodulators (Fig. 7, Fig. 8) (Shi et al., 2020; Zhang et al., 2020).

Corticosteroids decrease the host lung inflammatory response, thereby, reduce the risk of ARDS development. The success of corticosteroids depending on severity of COVID-19 was clinically proven with dexamethasone. The RECOVERY Collaborative Group conducted a 6425-patient, randomised controlled, open-label trial investigating the effect of dexamethasone on 28-day mortality rate (Group, 2021). The trial demonstrated the mortality rate in the dexamethasone-treated group depended on whether patients were receiving mechanical ventilation at randomisation. Mortality rate was significantly lower among patients receiving invasive mechanical ventilation (29% vs 41%; *P* ​< ​0.05) and oxygen through non-invasive mechanical ventilation (23% vs 26%; *P* ​< ​0.05) treated with dexamethasone than standard care, but not in patients without respiratory support (18% vs 14%; *P* ​> ​0.05) (Group, 2021). This led to guidelines in the UK, Europe and US being revised to recommend the use of glucocorticoids only in hospitalised COVID-19 patients requiring oxygen support (Johnson and Vinetz, 2020).

Cytokines, chemical messengers that induce the innate and adaptive immune response, can be divided into pro-inflammatory and anti-inflammatory (Dinarello, 2000). Cytokine storm, the uncontrolled release of pro-inflammatory cytokines, in the presence of HCoV leads to acute lung injury, ARDS and possible death (Zhang et al., 2020), with SARS-CoV-2 severity and mortality being associated with high cytokine levels (Saha et al., 2020). Therefore, treating the cytokine storm through cytokine production inhibitors, and other immunomodulators, could treat the HCoV-induced ARDS (Mehta et al., 2020). Interleukin-6 (IL-6), a pro-inflammatory cytokine, is strongly involved in inducing the cytokine storm and dysregulated inflammation associated with high HCoV mortality (Saha et al., 2020; Xu et al., 2020). Tocilizumab, a human recombinant IL-6 monoclonal antibody approved by the FDA for severe life-threatening cytokines release syndrome and rheumatic diseases (Zhang et al., 2020), has been repurposed against SARS-CoV-2, to bind to membrane-bound and soluble IL-6 receptors, inhibiting both IL-6 signalling pathways related to cytokine storm induction (Saha et al., 2020) (Fig. 7). Tocilizumab is included in the Chinese National Treatment Guidelines for severe COVID-19 cases (He, 2020) and was recommended in treatment of COVID-19, in combination with dexamethasone, by the US COVID-19 Treatment Guidelines Panel in March 2021 due to the promising preliminary results of the Randomised, Embedded, Multifactorial Adaptive Platform Trial for Community-Acquired Pneumonia (Gordon et al., 2021) and the open-label RECOVERY trials (Horby et al., 2021). Whilst tocilizumab was repurposed through observation (Saha et al., 2020), other cytokine storm inhibitors have been identified through screening methods, with sirolimus (also known as rapamycin) being one such example. Like tocilizumab, sirolimus is an immunosuppressant, hypothesised to control CoV cytokine storm, identified by a network-based drug repurposing model developed by Zhou et al. (2020b).

Fluvoxamine, an FDA approved drug initially used to treat obsessive-compulsive disorder, is an SSRI which was evaluated against SARS-CoV-2 (Lenze et al., 2020; Sukhatme et al., 2021) and is associated with reduced cytokine storm and inflammatory responses (Hoertel et al., 2021a, Hoertel et al., 2021b). In clinical studies, fluvoxamine has been shown to significantly reduce COVID-19 deterioration (Hashimoto et al., 2021; Lenze et al., 2020; Seftel and Boulware, 2021; Reis et al., 2021), however, like other SSRIs, it's mode of action against SARS-CoV-2 could be attributed to a multitude of other factors, including disrupting the endolysosomal trafficking, and interfering with ASM (Sukhatme et al., 2021).

IFNs are a group of anti-viral cytokines that regulate the innate immune system by intercell communication against pathogens and changing host gene transcription. IFNs have been shown to play a crucial role against HCoVs, controlling viral replication after infection and decreasing the production of type I IFNs which cause the dampening of the host immune response associated with HCoV severity and heightened mortality (Cherian et al., 2020; Mosaddeghi et al., 2020). Therefore, IFN therapy administration may be a promising HCoV treatment. A range of IFNs alone and in combination have shown *in vitro* inhibition of SARS-CoV, MERS-CoV and SARS-CoV-2 (Mosaddeghi et al., 2020). Chan et al. (Chan et al., 2013) conducted a MERS-CoV inhibition assay on known compounds which, amongst others, identified IFN-β1b with *in vitro* activity against MERS-CoV infected Vero cells at concentrations 3–4 times lower than those achievable in therapy (17.64 ​μM EC_50_; 3125 ​μM CC_50_), whose activity was enhanced by combination with mycophenolic acid. Sheahan et al. (2020) agreed with these findings, reporting potent *in vitro* inhibition of MERS-CoV by IFN-β (175 IU/mL EC_50_). IFN-β is reported to be the most potent IFN against MERS-CoV *in vitro* (Sheahan et al., 2020). IFN-β has also shown promising effects against SARS-CoV, with a screen of commercial antiviral agents against SARS-CoV conducted by Chen et al. (2004) identifying IFN-β1a and IFN-α. However, both interferons only gave efficacious *in vitro* activity (19.5 IU/mL EC_50_ for both IFN-β1a and IFN-α in Vero E6 cells) if incubated with cell lines prior to viral inoculation. Clinical comparison of IFN-β1a to placebo against COVID-19 yielded sufficiently promising results for the initiation of a phase 3 trial (Monk et al., 2021).

An alternative to the administration of recombinant IFNs is that of an IFN inducer, such as nitazoxanide, FDA-approved as an antiprotozoal against *Cryptosporidium parvum* intestinal infections with broad-spectrum antiviral potential (Rossignol, 2014, 2016). Nitazoxanide is also known to inhibit expression of MERS-CoV nucleocapsid (N) protein and suppress pro-inflammatory cytokine production, including IL-6 (Rossignol, 2016). Nitazoxanide was tested against CoVs by Cao, Forrest and Zhang (2015) who screened the 727 compounds of the NIH Clinical Collection against murine CoV infected cells, identifying nitazoxanide as one of the top 3 CoV inhibitors. Potent *in vitro* activity of nitazoxanide has been reported against MERS-CoV infected LLC-MK2 cells (0.92 ​μM IC_50_) at concentrations easily achievable in humans with nitazoxanide extended-release tablets (Rossignol, 2016). This potent *in vitro* activity is also seen against SARS-CoV-2 infected Vero E6 cells (2.12 ​μM EC_50_; 35.53 ​μM IC_50_), recommending further evaluation of nitazoxanide anti-CoV effects *in vivo* (Wang et al., 2020). Clinical studies of nitazoxanide treatment against COVID-19 do not illustrate convincing effectiveness (Mendieta Zerón et al., 2021; Rocco et al., 2021; Silva et al., 2021).

## 6 Conclusions

Whilst the majority of drugs trialled against COVID-19 yielded limited to no clinical benefit, the drug repurposing approach has identified some promising anti-HCoV therapies; whether that be by observation, high throughput screens, *in silico* modelling, and, in more current cases, a network-based AI approach. This was achieved much more quickly than conventional *de* novo drug design. Some of these agents are already being used as part of the standard of care in the clinic, while others are being evaluated in clinical trials. Unsurprisingly, the promising repurposed drugs identified were largely originally developed against other viruses, such as influenza, HIV and RSV, with arguably the most successful anti-HCoV compound to date, remdesivir, being originally developed for HIV, targeting RdRp. Of the targets explored, RdRp has received the most attention. This is likely due to the absence of the protein in humans, theoretically significantly reducing host toxicity. It is expected that many repurposed drug candidates will offer new chemical scaffolds that can be used to generate more potent and targeted inhibitors of HCoV in the next few years. From a therapeutic point of view, a combination approach using repurposed drugs appears to yield the best outcomes, as it can overcome the lack of drug optimisations against HCoV specifically. Due to the significant success of glucocorticoid, dexamethasone, and the cytokine storm inhibitor tocilizumab, we anticipate an increasing focus on the importance of combining antivirals with anti-inflammatories and/or immunomodulators to regulate the host immune response and the disease caused by HCoVs responsible for the viruses’ severity and mortality. Therefore, we foresee treatments to primarily center around combinatorial therapy with an immune response component as well as the pursuit of synergistic/additive compounds with anti-viral effects, facilitated by ever improving computation.

## 7 Outlook

As computational power has advanced, so too have drug repurposing strategies, moving from physical observation and high throughput cell-based screens to *in silico* modelling and virtual screening, saving time and valuable resources by streamlining the process. The COVID-19 pandemic has provided a wealth of new information about finding new drugs when a treatment is not available. The rapid integration of drug screening with clinical evaluation of promising repurposing drug candidates offers a new model for therapeutic drug development in pandemic situations. We predict the next step in drug repurposing techniques, already beginning to be seen, will be the normalisation of AI-inference modelling and advanced algorithm development, applying network science to disease prevention and treatment by examining patterns in biomedical data, taking a holistic view on drug-target interaction with regards to the disease system and host interactome, producing knowledge graphs to assist and accelerate drug development. The establishment of this AI-driven drug repurposing approach will be invaluable in the response to future pandemics.

## Ethic approval and consent to participate

Not applicable.

## Consent for publication

Not applicable.

## Competing interests

The authors declare no competing interests.

## Funding

This review did not receive any specific grant from funding agencies in the public, commercial, or not-for-profit sectors.

## CRediT authorship contribution statement

**Poppy O. Smith:** Writing – original draft, Writing – review & editing. **Peiqin Jin:** Artwork, Writing – review & editing. **Khondaker Miraz Rahman:** Writing – review & editing, Supervision.

## Declaration of competing interest

The authors declare that they have no known competing financial interests or personal relationships that could have appeared to influence the work reported in this paper.
